# Advances in Neurorehabilitation: Strategies and Outcomes for Traumatic Brain Injury Recovery

**DOI:** 10.7759/cureus.62242

**Published:** 2024-06-12

**Authors:** Purvi Kaurani, Ana Vitoria Moreira de Marchi Apolaro, Keerthi Kunchala, Shriya Maini, Huda A F Rges, Ashley Isaac, Mohit Lakkimsetti, Mohammed Raake, Zahra Nazir

**Affiliations:** 1 Neurology, DY Patil University School of Medicine, Navi Mumbai , IND; 2 Medicine, Faculdade de Medicina Santa Marcelina, São Paulo, BRA; 3 Internal Medicine, Sri Venkateswara Medical College, Tirupati, IND; 4 Medicine and Surgery, Dayanand Medical College and Hospital, Ludhiana, IND; 5 Mental Health, National Authority for Mental Health and Psychosocial Support, Benghazi, LBY; 6 General Medicine, Isra University Hospital, Hyderabad, PAK; 7 Internal Medicine, Mamata Medical College, Khammam, IND; 8 Surgery, Annamalai University, Chennai, IND; 9 Internal Medicine, Combined Military Hospital, Quetta, PAK

**Keywords:** virtual reality simulation, axonal injury, neurorehabilitation neurorehab, concussion, traumatic brain injury

## Abstract

Traumatic brain injury (TBI) consists of an external physical force that causes brain function impairment or pathology and globally affects 50 million people each year, with a cost of 400 billion US dollars. Clinical presentation of TBI can occur in many forms, and patients usually require prolonged hospital care and lifelong rehabilitation, which leads to an impact on the quality of life. For this narrative review, no particular method was used to extract data. With the aid of health descriptors and Medical Subject Heading (MeSH) terms, a search was thoroughly conducted in databases such as PubMed and Google Scholar. After the application of exclusion and inclusion criteria, a total of 146 articles were effectively used for this review. Results indicate that rehabilitation after TBI happens through neuroplasticity, which combines neural regeneration and functional reorganization. The role of technology, including artificial intelligence, virtual reality, robotics, computer interface, and neuromodulation, is to impact rehabilitation and life quality improvement significantly. Pharmacological intervention, however, did not result in any benefit when compared to standard care and still needs further research. It is possible to conclude that, given the high and diverse degree of disability associated with TBI, rehabilitation interventions should be precocious and tailored according to the individual’s needs in order to achieve the best possible results. An interdisciplinary patient-centered care health team and well-oriented family members should be involved in every stage. Lastly, strategies must be adequate, well-planned, and communicated to patients and caregivers to attain higher functional outcomes.

## Introduction and background

Characterized by a disruption in brain function or the manifestation of brain pathology due to an external physical force [1], 50 million people are affected with traumatic brain injury (TBI) annually worldwide. This staggering figure suggests that roughly half of the global population will experience such an injury during their lifetime [2]. In the UK, TBI is the primary cause of death and disability among people under 40 [3]. Additionally, lower-income and middle-income countries tend to experience even higher rates of morbidity and mortality associated with TBI. Annually, TBI places a significant economic burden on the global economy, costing approximately 400 billion US dollars and representing 0.5% of the gross world product [2]. TBI, especially when it is more severe (such as moderate-to-severe cases), has been recognized as a contributing factor to the development of all-cause dementia and Parkinson’s disease (PD) [3]. However, the risk for specific subtypes of dementia varies.

The classification of injury severity involves assessing factors such as level of consciousness, amnesia status, and neuroimaging findings. The Glasgow Coma Scale (GCS), a widely used tool, assesses consciousness through eye-opening, verbal response, and motor response measures, utilizing a scale ranging from 1 to 15 [4]. Additional metrics, less frequently utilized, encompass parameters such as time taken to follow commands, loss of consciousness, and post-traumatic amnesia [5].

Rehabilitation focuses on gaining a lost function. It promotes recovery through neuroplasticity to develop lost function, reducing complications, and improving quality of life. Although many guidelines for TBI rehabilitation are limited, evidence-based research has begun to surface and is maturing [6-8]. Recent research indicates a negative correlation between prolonged intervals from discharge from intensive care to admission to rehabilitation and unfavorable outcomes, not solely attributed to initial injury severity [9]. Additionally, patients benefit from a more favorable outcome when rehabilitation is seamlessly integrated as part of a continuous process initiated in the intensive care unit [10]. Cognitive rehabilitation (CR) denotes the implementation of interventions aimed at compensating for or resolving cognitive impairments, ultimately reducing disability [11]. CR is typically recommended for managing the aftermath of moderate to severe TBI. However, repetitive concussions or mild TBI (mTBI) may lead to enduring cognitive issues in specific patient subsets. While transient cognitive dysfunction, such as difficulty concentrating or memory problems, is prevalent during the initial two to four weeks following a sports-related concussion and can persist for up to three months after non-sports-related mTBI, these effects typically resolve without the need for CR in the majority of athletes and non-athletes [12].

## Review

TBI can present in many forms, such as concussions, brain contusions, brain hemorrhages, diffuse axonal injuries, coup, contrecoup, and coup-contrecoup brain injuries, penetrating brain injuries, and second impact syndrome [4,13]. The primary underlying mechanism of TBI causing neurological impairment is the blood-brain barrier (BBB) disruption [14]. The outcome of the neurological injury depends on different mechanisms/stages. Primary insult occurs at the time of injury; this type of injury is mainly sensitive to preventive but not therapeutic interventions. Secondary insult represents consecutive pathological processes initiated at the time of injury with delayed clinical presentation, and these types of injury are sensitive to therapeutic interventions [15].

TBI also includes unique clinical presentations and phenotypes that rely on pre-, post-, and damage-related factors. Age, sex, genetics, pre-existing conditions, and injury-related variables (such as the extent of the injury and comorbidities) are among the TBI phenotypes. Recurring symptoms can eventually lead to negative consequences and varying recovery routes [16]. After prolonged hospital care for severe TBI, they require long-term rehabilitation and may have long-term physical, cognitive, and psychological disorders. Such disorders affect the quality of life (social and economic) [17].

Principles of neurorehabilitation in TBI 

Neuroplasticity, sometimes called neural plasticity or brain plasticity, is a process in which the brain adapts its structure and functions [18]. The best term to describe this concept is "the nervous system's capacity to reorganize its structure, functions, or connections in response to internal or external stimuli." In clinical practice, this refers to the brain's ability to adapt following a stroke or TBI [18,19].

Two main processes underlie neuroplasticity, which include neural regeneration and collateral sprouting. This encompasses ideas such neurogenesis and synaptic plasticity [10,12,18,19]. Functional reorganization encompasses ideas such as diaschisis, variation, and equipotentiality [18,20].

Goals of rehabilitation after TBI

**Figure 1:**
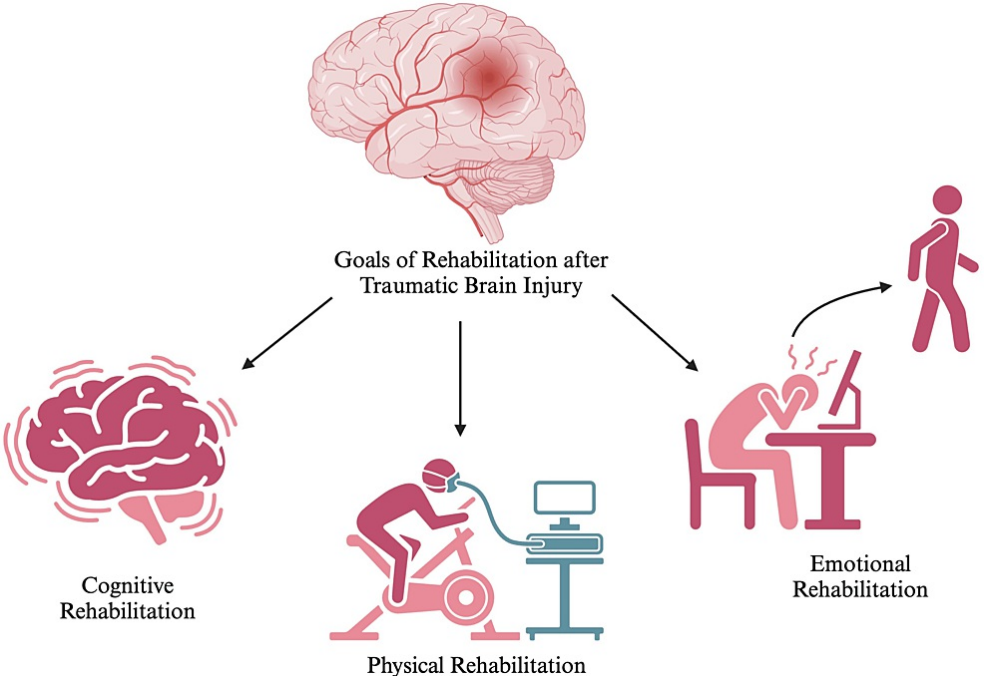
Goals of rehabilitation after traumatic brain injury Source reference: [19,20]. Created using biorender.com. This figure is the original work of the authors.

Figure 1 highlights the goals of rehabilitation after TBI.

A systematic, functionally focused program of treatment activities based on evaluation and comprehension of the patient's cognitive and behavioral deficiencies is known as cognitive rehabilitation [19]. It can also include the use of compensatory processes to establish alternative patterns of cognitive activity (through compensatory cognitive mechanisms for impaired neurologic systems) or create new patterns of activity through external support devices (e.g., prosthesis or adaptive aids) [19]. While not explicitly focused on modifying cognitive impairments directly, these interventions aim to improve the patient's overall functioning and quality of life [19]. Restorative interventions are limited to directly addressing the cognitive disturbances caused by brain damage.

Recent studies found that physical exercise promoted cerebral angiogenesis, vasomotor reactivity, and neurotrophic factor release but also reduced apoptosis processes, excitotoxicity, and inflammation around the infarct and could improve the regulation of motor unit activation [20]. However, physical training is insufficient to restore neural and motor function fully. A recent approach to stroke treatment is to combine exercise with pharmacological treatments known to increase and accelerate neuroplasticity [20]. In this review, we discuss the effects of physical training with or without adjunctive drug therapy on neuroplasticity and motor recovery after stroke. Although regular exercise reduces the risk of stroke, it can occur in physically active people. However, the endogenous neuroprotective effects induced by pre-stroke physical activity can induce recovery, reducing both brain damage and the severity of motor outcomes [20].

Except for studies of individuals with psychiatric disorders associated with abnormal emotion regulation, such as schizophrenia, major depression, bipolar disorder, posttraumatic stress disorder, anxiety, and panic disorders, few studies have examined the effects of emotion in conditions such as neurotrauma. Alternatively, neurodevelopmental disorders that affect cognitive and sensorimotor domains [21]. A vital characteristic of the central nervous system is that it adapts to complex changes, including those caused by injury. Inherent plasticity contributes to at least some functional recovery. However, spontaneous repair mechanisms are rarely sufficient to support a significant long-term recovery, whereas post-injury experience can be a potent modulator of functional recovery [21]. In recent years, it has become clear that environmental enrichment strongly influences neuroplasticity and neurological recovery [21]. Its beneficial effects have been demonstrated in various experimental models of brain diseases, including enhanced cognitive function, delayed onset of neurodegenerative diseases, stimulation of the synthesis of neuromodulators, and increased cellular and molecular plasticity that influence arousal levels, motivation, attention, attachment, and emotions (such as noradrenaline, acetylcholine, dopamine, and serotonin) [21].

Neuromodulation mechanism

Neuromodulation encompasses invasive and non-invasive interventions that alter neuronal activity or excitability [22]. Along with acute changes, neuromodulation also leads to persistent changes in neural function and connectivity; that is, it produces neuroplastic changes and, therefore, can be used to reverse maladaptive neuroplastic changes already occurring in the brain or to prevent maladaptive neuroplastic changes. Alternatively, it enhances adaptive neuroplastic changes in the brain, such as during functional recovery after central nervous system (CNS) lesions [22]. Many neuromodulation methods are in various preclinical and clinical testing stages or fully implemented in medical practice [22].

Functional plasticity has long been associated with development; however, such mechanisms may also be important in the adult nervous system [23]. Postmitotic neurons, once believed to be structurally and characteristically stable in the brain and spinal cord, were later found to undergo significant adaptations in neuronal activity. These adaptations included changes in morphological characteristics, gene expression, and synaptic strength [23]. Furthermore, neuronal firing behavior has influenced many mechanisms ranging from synaptic plasticity to dendritic arborization and even neuronal regeneration [23]. Such mechanisms are driven by internal signaling molecular factors that have been increasingly elucidated in recent decades. Several intrinsic and extrinsic factors have direct activity-related expression changes, brain-derived neurotrophic factor (BDNF), and neuronal PAS domain protein 4 (Npas4), exhibit activity-related expression changes and significantly impact neuronal expression [23]. Recent literature describes the regulatory mechanisms of gene expression associated with neuronal activity, including the potential role of activity-induced double-stranded DNA breaks and RNA splicing, demonstrating the depth and breadth of the complexity of activity-related gene expression [23,24]. Understanding how neuronal activity affects gene expression is critical to developing potential therapies that modulate neuronal activity.

Rehabilitation strategies for TBI

Early Intervention and Acute Care Strategies

TBI causes debilitating impairments that affect the physical, cognitive, speech, and executive functions. Patients with TBI have a poor quality of life due to behavioral disturbances, emotional and social deterioration, and physical disabilities. Rehabilitation measures play a crucial role in preventing and reducing the burden of such impairments in patients with TBI.

Early interventions in the management of TBI begin with first responders, such as emergency medical services personnel and paramedics, evaluating and stabilizing the patient at the scene of the injury before the patient is brought to a hospital setting [25]. Initial assessment involves monitoring vital signs and oxygen saturation and calculating the Glasgow coma scale (GCS) scores [25]. Upon arrival, the patient is managed according to Advanced Traumatic Life Support (ATLS) steps, which are similar to the protocol applied to all other trauma patients [26].

The most critical steps are maintaining the airway, ventilation, and arterial pressure. A randomized multicenter trial that included more than 70% of patients with TBI demonstrated improved survival outcomes in patients with GCS ≤ 8 when hypertonic saline was used over Ringer’s lactate [27]. Tracheal intubation is associated with better functional outcomes in patients with a GCS score of less than 10 [27,28]. This finding is also by a study conducted by Davis et al. [29], which illustrates that the effects of intubation in patients with low GCS scores are likely to be more beneficial than harmful [29]. The GCS is an assessment tool for patients with impaired consciousness [29].

After initial evaluation, specialists perform a complete neurologic examination, including imaging. Given the intensity of the injury, the patient is further managed in acute care settings, such as intensive care units or specialist neurocritical care units. Further specialist neurocritical care interventions are targeted at controlling intracranial pressure (ICP) and cerebral perfusion pressure (CPP) through protocol-driven therapy [30,31]. Measures include providing supplemental oxygen or mechanical ventilation depending on the degree of airway compromise, hyperosmolar therapy, and early enteral nutrition [32].

A critical intervention following TBI is physiotherapy, which must be initiated during the initial phase of patient admission and continued further even after discharge. According to the INCOG guidelines, based on the degree of fatigue, confusion, and agitation in patients with PTA, physical therapists must provide therapy tailored to individualized needs while maintaining flexibility in session length, intensity, and location [33].

Techniques for physiotherapy might include early mobilization via passive- or active-assisted handling, as advised by the physiotherapists, positioning the patient on the bed in different positions, including side-lying and prone when appropriate, and changing the position every two hours, as well as assistance in getting out of bed via a wheelchair or specialist supportive chairs to enhance early recovery [34,35]. Aerobic exercise can also be implemented as a rehabilitation method as it promotes cardiovascular fitness and improves physical health [36]. In addition, it has shown improved outcomes in cognitive functions [37] and decreased levels of depression [38]. Walking and jogging, typically done on a treadmill, is the most applied aerobic intervention in patients with varying injury severity [39,40].

Cognitive impairments are the most debilitating sequelae of TBI. Attention disorders, memory deficits, and disturbances in executive functions are the most commonly encountered cognitive deficits of TBI at all levels of severity [41]. A detailed neuropsychological assessment to evaluate the patient’s cognitive abilities is conducted prior to the initiation of cognitive rehabilitation [42]. Cognitive rehabilitation interventions are structured activities tailored to the patient’s needs. The two broad cognitive rehabilitation approaches are divided into compensatory and restorative strategies [43]. Compensatory approaches (internal and external) are designed to compensate for functional impairments by providing alternative strategies for carrying out daily activities. Restorative methods are targeted at directly strengthening and restoring impaired cognitive functions through repetitive activities [42].

Rehabilitation interventions for memory deficits involve both compensatory and restorative approaches. Performing daily tasks, such as remembering to take medications on time or locking the house when going out, is associated with prospective memory [44]. Prospective memory can be rehabilitated through external compensatory strategies such as a notebook to write down important tasks, a phone calendar to remember important dates, and assistive technology such as pagers, voice recorders, and smartphones [45]. Internal compensatory strategies include visual imagery, repeated practice, retrieval practice, and metacognitive strategies [45]. However, the benefit of internal strategies was found to be short-term, especially in patients with chronic TBI [46]. 

Attention deficits include difficulties in concentration, delayed reaction time, decreased processing speed, distractibility, and inability to multitask. Attention process training (APT) is a direct training program designed to improve visual and auditory attention [47]. This specific skilled training program targets five components of attention: focused, sustained, selective, alternating, and divided attention [47]. The training program begins with simple tasks that require less attention and progresses to complex tasks that demand increasing attention spans. A result from RCT demonstrated that patients with mild to moderate brain injury who received a combination of cognitive remediation (including a memory notebook and direct attention training) and cognitive behavioral therapy performed better in divided auditory attention compared to the control group. They also showed improved emotional stability [48]. Moreover, individuals with TBI can be trained to learn two different activities that are essential on an everyday basis, such as walking and talking. This will increase the span of divided attention and lead to functional improvements in performing essential tasks.

Deficits in executive functioning highly impact the person’s social, academic, personal, and professional life. Given that executive impairments are among the most difficult to treat among all other cognitive deficits, it is necessary to develop optimized and systematic intervention therapies. Metacognitive strategies are proven more effective than conventional rehabilitation techniques [49] as they aim to improve self-awareness of one’s actions and behaviors through self-evaluation. The strategies include self-prediction and performance monitoring in goal-directed activities and self-discovery of errors in performance [50,51]. Metacognitive strategies should be created using relevant daily activities to improve functional outcomes. Individuals should be encouraged to self-monitor their performance through verbal and video feedback, allowing the patients to identify and address their errors in future tasks. An RCT conducted by Schmidt et al. examined three types of feedback: video and verbal, verbal-only, and experiential feedback only. Video and verbal feedback was superior to the other two types of feedback in terms of increased self-awareness about deficits in performance activities [50-52].

Challenges in speech and communication are common in TBI, following social cognition impairments. This results in communication disorders that profoundly impact the patient’s social interactions. Furthermore, difficulty articulating and expressing one’s thoughts, emotions, and ideas lowers the patient’s morale and confidence. Intervention strategies to improve the ability to communicate revolve around multiple variables, such as the severity of the injury, pain, emotional dysregulations, and patient environment. Speech and language therapy, including constraint-induced aphasia therapy (CIAT), computer-assisted therapy, melodic intonation therapy, and neurostimulation techniques such as transcranial direct current stimulation (TDCS), have been found to improve dysarthria and aphasia in patients with brain injury. In addition, the rehabilitation team should ensure that the language known to and comfortable by the patient should be used. The role of the family plays a more significant part in overcoming these challenges as patients may be more comfortable interacting with family members than with healthcare professionals [53-56].

**Figure 2:**
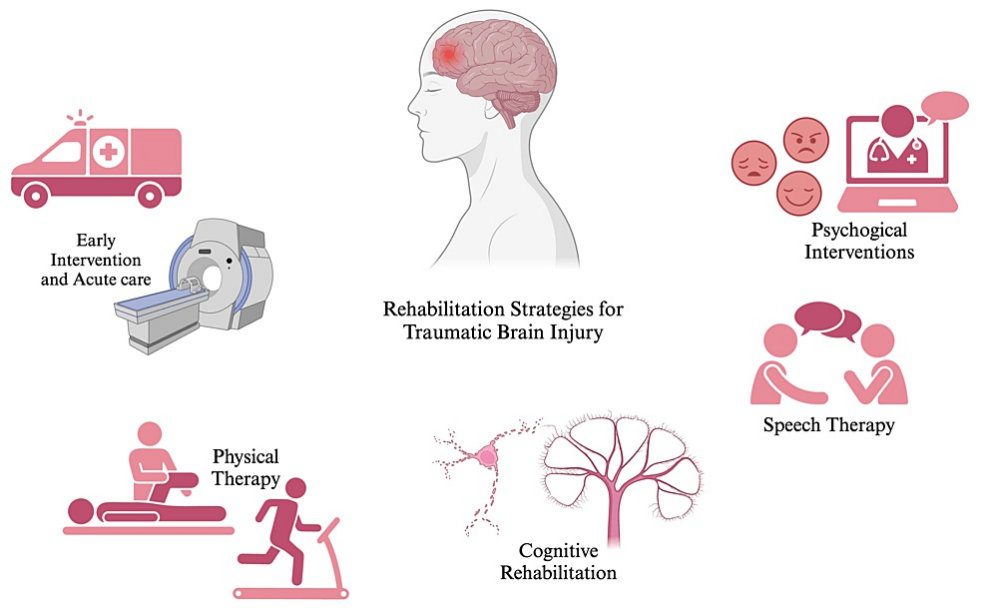
Rehabilitation strategies for traumatic brain injury Source reference: [62-64]. Created using biorender.com. This figure is the original work of the authors.

Following TBI, patients usually develop behavioral and emotional disturbances, such as depression, irritability, anxiety, and anger [57]. Holistic psychotherapy, a technique designed explicitly for TBI patients, focuses on strategies to recognize the disability and work on it in a progressive manner [57]. Cognitive behavioral therapy is the most preferred approach of psychotherapy for the improvement of psychological disturbances. CBT is a strategy to enhance cognition directly associated with improved mood and state of mind [58]. Furthermore, music-based cognitive therapy has shown promising results in enhancing patient’s mood and alleviating anxiety and depressive symptoms [59]. Besides the soothing effects of music on the individual’s emotions, it also nurtures muscle memory and attention span. Research has also demonstrated the potential efficacy of mindfulness training in the improvement of depressive symptoms and PTSD-associated symptoms [60]. This training employs gentle yoga, meditation, mindful eating, and walking to help focus the mind on a single point of reference, such as the breath or an object. During the practice, it encourages acknowledging thoughts or emotions that arise without judgment [61]. Figure 2 presents the rehabilitation strategies for TBI.

Innovative approaches in TBI rehabilitation

Role of Technology

Technology and virtual reality (VR), whether immersive or non-immersive, have played a vital role in neurorehabilitation in patients after a TBI. Multiple RCT studies included using VR in patients after a stroke to improve balance, mobility, and gait in stroke rehabilitation inpatients, which concluded that VR exercises in inpatient stroke rehabilitation improved their mobility-related outcomes when measured by a two-minute walk test and time up and go test, in addition to the Chedoke-McMaster Leg domain measurement, showing reduced impairment in the lower extremity [62]. A four-week intervention consisting of 12 treadmill training sessions with VR improved balance and mobility in people with TBI more effectively than treadmill training alone or the standard of care [63]. Outcomes were measured using primary and secondary measures, and as a primary measure, it included recruitment, retention, and enrolment rates, tolerance to intervention, adverse events, and completeness of outcome measures. A 10-meter walk test, community balance, mobility scale, timed up and go, and six-minute walk test were the secondary outcome measures. However, in a home-based exercise program (HEP) that utilized a VR gaming system in a 12-week physical therapy (PT) intervention in TBI individuals, it was found that VR training was not more beneficial than a traditional home-based exercise program for improving balance [64]. The community balance and mobility scale (CB&M) is a primary measure of outcomes, and the activities-specific balance confidence scale (ABC), balance evaluation systems test (BESTest), and participation assessment with recombined tools-objective (PART-O) were the secondary measures of outcomes.

In contrast to treatment as usual alone, elements virtual rehabilitation combined with treatment as usual significantly improved motor and cognitive function in individuals recovering from stroke through exploratory and goal-directed upper-limb movements [65]. In order to measure cognitive outcomes, the Montreal Cognitive Assessment was used, and selected CogState subtests were used. The box and blocks test was used to measure motor upper limb skills, and the neurobehavioral functioning inventory was used to measure everyday participation. Moreover, improvements were maintained at the one-month follow-up, as shown by the experimental group. It has been demonstrated that a four-week no-immersion VR intervention improved the performance of adults with TBI on digit symbols [66], verbal and visual learning tasks, and reaction and movement times when assessed with attention, learning, memory, information processing, and reaction times. There were improvements in reaction times and movement times after a single round of VR exercise.

When considering how TBI affects executive function and people's ability to lead independent lives in terms of daily life activities, some pilot randomized controlled trials with VR exercises that simulate everyday life were implemented using VR supermarkets (VMall) [67]. Outcomes were measured using the multiple errands test-simplified version (MET-SV) and the executive function performance test (EFPT). Results showed more significant improvement in complex everyday activities in VR therapy compared to cognitive retraining occupational therapy without VR.

Another simulation is the Neurocognitive Driving Rehabilitation in Virtual Environments (NeuroDRIVE) pilot randomized controlled trial (RCT) that significantly improved two skills: working memory and visual search/selective attention [68]. However, by comparison, there is no significant change in the untrained cognitive areas, neurobehavioral symptoms, or driving skills, suggesting that immersive VR can achieve some cognitive rehabilitation goals when matched to VR training exercises.

In another feasibility study using VR driving simulation rehabilitation training (VRDSRT) with military personnel, driving performance was significantly improved with reduced road rage and risky driving in post-assessment. The purpose of those VR simulations is to provide an approach that integrates cognitive and executive functions with ecological validity [69]. Similarly, in an outpatient military hospital clinic, BrightBrainer Virtual Rehabilitation (BBVR) was used as a means to provide integrative and intensive VR rehabilitation to US soldiers suffering from TBI and cerebrovascular accident (CVA). In a compact, adaptive VR system, the BBVR system combines cognitive and physical training that utilizes bilateral training, which has been found to promote improved motor functioning for people who have experienced ABI above and beyond unilateral training [60,70,71]. Seven clinical outcomes included the Fugl-Meyer assessment of upper extremity, box and blocks test, Jebsen-Taylor hand function test, automated neuropsychological assessment metrics, neurobehavioral symptom inventory, a quick inventory of depressive symptomatology-self-report, and post-traumatic stress disorder checklist - civilian version as the majority of patient with males and mild TBI constituted most of the patients. However, TBI and CVA were included. At baseline, participants had current comorbid psychological symptoms, persisted neurobehavioral symptoms, and experienced regular headaches. The majority of participants reported cognitive deficits, including speech difficulties and post-traumatic amnesia. These findings were based on participant endorsement and electronic medical record review. Both participants and providers found the BBVR system easy to use and were satisfied. Although cognitive function improved with the use of the BBVR system, other symptoms did not improve significantly. In an outpatient military setting, the BBVR system can be used to treat symptoms of acquired brain injury (ABI). According to the researchers, BBVR's clinical effectiveness should be further investigated in a randomized trial.

In efforts to improve sociocognitive deficit and impaired social competence in children with acquired brain injury using VR technology, a rehabilitation design made of interactive computer-based applications was implemented using Multitouch-Multiuser Tabletop (MMT) devices, Diamond Touch Table (DTT), and Snowflake MultiTeach (MT) plus MediqVR VR platform [72]. Results suggested improvement in children's executive and cooperative skills with Snowflake MT rehabilitation; new communication and language skills, metacognitive skills, and coping with difficult social situations were developed with DTT. In rehabilitating children with TBI to improve executive function, a VR-based interactive cognitive system (VICT) was used in an RCT. It has shown that it is an intervention with potential efficacy in moderate to severe TBI and its safety. The intervention group reported adequate VR satisfaction, low levels of physical exertion, and simulator sickness [73].

The study's findings focused on improving attention, memory, executive function, and problem-solving skills after a TBI. Artificial intelligence 3D VR vocational problem-solving, such as the Tower of London Test, Wisconsin Card Sorting Test, and Vocational Cognitive Rating Scale, compared with conventional psycho-educational methods, showed that people with mild cognitive impairment showed better brains that could choose and prioritize specific memories over others and better awareness of their memory abilities [74].

TBI can cause cognitive impairment, which can be disabling. Furthermore, depressive symptoms are partially mediated by cognitive impairment after TBI, resulting in a decreased health-related quality of life [75]. In terms of robotic-assisted rehabilitation training in TBI individuals, a review evaluated a couple of innovations, such as self-feeding robots, which have been found to improve the quality of everyday life and the application of collaboration. Additionally, the application of the wheelchair assistant system (CWAS) included people with TBI and cerebral palsy who were trained to use it with and without path guidance, and only after a few training sessions, the results show their ability to use the CWAS safely and efficiently even in an environment with obstacles and narrow passageways, which provided driving assistance and providing an increase in mobility [76,77].

Furthermore, in a case study of a 24-year-old female with TBI along with severe diffuse axonal injury, the use of robotic assistive gait technology during a six-month inpatient rehabilitation may be beneficial when combined with conventional therapy in reducing disablement, as it can help increase repetition and cardiovascular activity during rehabilitation. In children with TBI and central gait impairment, the use of robotic-assisted locomotor training has been successfully integrated into rehabilitation programs as it has shown remarkable results and a significant increase in the six-minute walking test, functional ambulation categories, and standing dimension of the gross motor function [78,79]. Moreover, in a randomized prospective study, the use of robotic-assisted treadmill training (RATT) in comparison with manually assisted treadmill training (MATT) in persons with TBI showed only improvement in symmetry of gait (step length) [80]. No significant difference was found in endurance, gait velocity, and stroke impact scale (SIS). Therefore, it was evident that the use of either MATT or RATT training benefits persons with chronic TBI in terms of improvement in gait parameters.

Visual feedback distortion rehabilitation for upper limbs in persons with TBI to support individuals unwilling to move beyond their current performance level has resulted in functional improvement beyond one's level [81]. To encourage the subjects to improve their performance, the range of movement was gradually increased in the visual feedback in a subtle way that prevented them from realizing that they were being asked to do better in order to avoid resistance to improvement. In chronic non-ambulatory acquired brain injury survivors, the Locomat and Walkbot robotic gait training combined with conventional gait-oriented physiotherapy [82]. A study conducted in the Republic of Korea over three years (2014-2017) found that combining the Locomat and Walkbot robotic gait training with conventional gait-oriented physiotherapy effectively improved the mobility of chronic non-ambulatory acquired brain injury survivors. The study was retrospective and cross-sectional. However, these devices did not differ significantly from one another. The Lokomat coordinates hip and knee joints and uses an ankle-foot orthosis to facilitate walking on a treadmill, which was the first gait orthotic to achieve this in gait-impaired patients. Furthermore, a retrospective case-control study suggested that including VR with Lokomat in the rehabilitation of persons with TBI results in the overall improvement in cognitive function, everyday well-being, and quality of life in addition to their awareness of their mental and physical state [83-85].

In conclusion, multiple methods of VR have been implemented for rehabilitation after TBI, and improvement in various aspects was noted in executive function, gait, and physical abilities to enhance the patient's ability for everyday function. Table 1 depicts various VR modalities for patients with TBI.

**Table 1:** Virtual rehabilitation intervention in adults with traumatic brain injury

VR Modality	Outcome Measurements	Outcomes	Duration
VR exercises [62]	Timed Up and Go and the Two-Minute walk test, in addition to the Chedoke McMaster Leg domain measurement	Improved their mobility and balance	4 weeks
Treadmill using VR [63]	Primary measure of outcome: recruitment, retention and enrolment rates, tolerance to intervention, adverse events, and completeness of outcome measures. Secondary measures of outcome:10-Meter Walk Test, Community Balance, and Mobility Scale, Timed Up and Go and 6-Minute Walk Test	Improved balance and mobility	4 weeks-12 session
Home-based exercise program (HEP) [64]	Primary outcome measure: Community Balance and Mobility Scale (CB&M). Secondary outcome measure: Activities-Specific Balance Confidence Scale (ABC), Balance Evaluation Systems Test (BESTest) and Participation Assessment with Recombined Tools-Objective (PART-O)	Not more beneficial than traditional home-based exercise program	12 weeks
Elements virtual rehabilitation combined with treatment as usual [65]	Motor measures of outcome: the Box and Blocks and exploratory and goal-directed upper-limb movements. Cognitive measure of outcome: Montreal Cognitive Assessment, CogState subtests, Neurobehavioral Functioning Inventory was used to measure everyday participation	Improved both motor and cognitive function in individuals recovering from stroke	4 weeks
No-immersion VR intervention [66]	Digit symbol, attention, learning, memory, information processing and reaction times	Improved the performance on verbal and visual learning tasks, reaction and movement times, after a single bout of VR exercise	4 weeks
Virtual reality (VR) supermarket (VMall) [67]	Outcomes were measured using Multiple Errands Test-Simplified Version (MET-SV), the Executive Function Performance Test (EFPT)	Improvement in complex everyday activities compared to cognitive retraining occupational therapy without VR	
Neurocognitive Driving Rehabilitation in Virtual Environments (NeuroDRIVE) [68]	Neuropsychological assessment. Virtual reality driving assessment	Significant improvement in two skills which are working memory and visual search/selective attention	6 sessions for 90 minutes in 4 weeks
Driving simulation rehabilitation training (VRDSRT) [69]	Pre- and post-assessment Simulator driving. Road rage and risky driving behavior	Significant improvement in driving performance with reduced road rage and risky driving	4-6 session for 60-9- minutes
BrightBrainer Virtual Rehabilitation (BBVR) [84]	Seven clinical outcomes included the Fugl-Meyer Assessment of Upper Extremity, Box and Blocks Test, Jebsen-Taylor Hand Function Test, Automated Neuropsychological Assessment Metrics, Neurobehavioral Symptom Inventory, Quick Inventory of Depressive Symptomatology-Self-Report, and Post Traumatic Stress Disorder Checklist - Civilian Version.	Cognitive function improved with the use of the BBVR system, other symptoms did not improve significantly	6 weeks
Artificial intelligence 3-D virtual reality vocational problem-solving [74]	Comparison of pre- and post-assessment of vocational problem-solving such as Tower of London, Wisconsin Card Sorting Test, and Cognitive Rating Scale	Improvement in selective memory and awareness of memory	

Table 2 highlights various robotic interventions in adults with TBI.

**Table 2:** Robotic interventions in adults with TBI

Robotic Modality	Outcome Measurements	Outcomes	Duration
Robotic assistive gait technology [78]		Maybe beneficial when combined with conventional therapy in reducing disablement, increase repetition and cardiovascular activity during rehabilitation	Six months
Robotic-assisted treadmill training (RATT) [80]	Stroke impact scale (SIS)	Only improvement in symmetry of gait (step length) and there no significant difference found in endurance, gait velocity and stroke impact scale (SIS). No advantage of robotic-assisted treadmill training (RATT) over manually assisted treadmill training (MATT)	
Bilateral robot-assisted mirror therapy (RMT) [85]	The Fugl-Meyer Assessment upper extremity (FMA-UE) motor score. Motricity Index (MI). FMA-UE sensation test	Great improvement and treatment efficacy on motor function	15 sessions 30 minutes each, 5 weeks

Visual feedback distortion rehabilitation for upper limbs in persons with TBI to support individuals unwilling to move beyond their current performance level has resulted in functional improvement beyond one's level. Moreover, to encourage the subjects to improve their performance, the range of movement was gradually increased in the visual feedback in a subtle way that prevented them from realizing that they were being asked to do better to avoid resistance to improvement. In chronic non-ambulatory acquired brain injury survivors, the Locomat and Walkbot robotic gait training combines conventional gait-oriented physiotherapy. A study conducted in the Republic of Korea over three years (2014-2017) found that combining the Locomat and Walkbot robotic gait training with conventional gait-oriented physiotherapy effectively improved the mobility of chronic non-ambulatory acquired brain injury survivors. The study was retrospective and cross-sectional. However, these devices did not differ significantly from one another. The Lokomat coordinates hip and knee joints and uses an ankle-foot orthosis to facilitate walking on a treadmill, which was the first gait orthotic to achieve this in gait-impaired patients. Furthermore, a retrospective case-control study suggested that including VR with Lokomat in the rehabilitation of persons with TBI results in the overall improvement in cognitive function, everyday well-being, and quality of life, in addition to their awareness of their mental and physical state.

Robotic wheelchairs with leg exoskeletons for exercising legs have been developed, and through pedaling exercises, people with lower leg disabilities can maintain their mobility while strengthening their limbs. An evaluation of exercise efficiency involving the gluteus medius muscles was conducted, and positive results were found. Therefore, individuals with lower leg disabilities can benefit from this technology because it combines assistive and rehabilitation functions.

A depth sensor worn on a belt is being explored to integrate environmental data into powered lower-limb prosthetic control systems. Because of the data sources' inherent inter- and intra-subject variability, the current systems that use electromyography (EMG), kinetics, and kinematics for prediction are vulnerable to substantial errors. The best results were achieved when Vision, EMG, inertial measurement unit (IMU), and goniometer were combined. Vision-based environmental data are expected to improve forward prediction accuracy in powered lower-limb prosthetics and exoskeletons.

Additionally, scientists are investigating selective sensing to enhance power control systems and wearable assistive devices. This approach allows sensors to detect environmental data without needing physical contact with the environment. Technology advancements have made this technology more feasible. These sensors are beneficial for predicting upper-limb grasp and arm endpoint control, segmenting gait events, predicting locomotion modes, and controlling lower-limb devices. Before their use outside the laboratory, these technologies are explored further.

Neurofeedback and brain-computer interfaces (BCI)

In recent years, implantable BCIs have provided neural recordings with increased spatial resolution, making it possible to record and decode neural signals more accurately. These recordings can be used to control functional electrical stimulation (FES) devices that stimulate the muscles directly, bypassing the damaged neural pathways. Combining these technologies has shown promising results in restoring functional movement in paralyzed patients.

Developing BCI-FES systems poses numerous challenges, including decoding neural signals into meaningful motor commands. Sophisticated algorithms can decode these signals, allowing FES devices to be precisely controlled. However, further research needs to be conducted in this area to improve the accuracy and reliability of these systems. In addition, a challenge exists in developing FES devices that mimic healthy individuals' movement patterns since the stimulation pulses must be precisely controlled in timing, intensity, and duration. Despite the advancements in electrode design and stimulation algorithms, it is still necessary to make further improvements to produce more naturalistic movements. It has been possible to restore lost function to individuals with disabilities by integrating BCI and FES technologies despite these challenges. These technologies may one day provide a viable solution to spinal cord injury, stroke, and TBI patients.

To assist patients with TBI in terms of communicating or mobilizing, in order to create BCIs, individuals with severe paralysis had to decode their intentions. It has been discovered that, using a BCI involves learning a new task, which may alter the brain's plasticity and learning mechanism. Machine learning algorithms that adapt to a user's brain activity can only be created after intensive training, and users can only use a BCI once they have received such training. The central nervous system learns through neuroplasticity, as does this training.

Individuals with impaired force control were found to be able to use noninvasive high-gamma neural brain-machine interface (nrBMI) to control force. The high-gamma modulation was observed in an electroencephalogram recorded over a hemicraniectomy (hEEG) but not skull-intact EEG. Compared to low-frequency neurorehabilitation, nrBMI control, and high-gamma control significantly improved timing synchronization between neural modulation onset and nrBMI output/haptic feedback. For brain injury recovery, improved synchrony between neural modulation and force control is critical to improve the ability of nrBMIs to induce plasticity in neural circuits. Individuals with impaired force control may benefit from high-gamma nrBMIs.

Emerging pharmacological treatments and their role in rehabilitation

Pharmacological intervention was mainly studied in acute settings in hospitalized patients. As there were not many drug therapies tested in a neuronal rehabilitation setting. However, some tested drugs showed neuroprotective properties, and some explored improving cognitive recovery.

Aside from its hormonal properties, progesterone is being studied for its role as a potential neuromodulatory agent. Progesterone receptors are expressed in various sites in the brain, with recent studies indicating that progesterone causes upregulation of receptors in the brain, in addition to regulation of neuronal excitability. Progesterone has anti-inflammatory and neuroprotective traits in experimental animals, reducing cerebral edema and ICP risk. However, no significant clinical value was found in clinical trials compared to placebo in patients with TBI [86-90]. More drugs have been tested in acute TBI settings, assuming their role as neuroprotective agents such as erythropoietin, which has only shown benefit in reducing mortality; no benefit was found in preventing poor outcomes.

As an M2 ion channel blocker in viruses and its dopaminergic role as a weak NMDA glutamate antagonist, amantadine has been found to impede the recovery of cognitive function if given within the first month. However, some conflicting results require further studies to assess the role of amantadine. One significant finding is that amantadine reduces the severity of aggression and irritability in TBI patients [91-96].

Botulism toxin is known for its role as an anesthetic agent that relaxes the muscle and enables its movement. Only a few pharmacological interventions were integrated into a rehabilitation program. One of the pharmacological interventions used in rehabilitation was Botulism toxin injections, which have been found to be effective in reducing muscle spasticity in those with stroke or TBI and improving muscle tone. 

There has been a limited number of studies regarding genetic interventions. However, some studies evaluated the ability to use microRNA as a genetic biomarker in diseases of consciousness and TBI. Downregulation of MMP-9mRNA and MMP-9 protein level expression was found with mild hypothermia therapy intervention in individuals with TBI [97-99].

Genetic therapeutic interventions in a large clinical cohort of stroke patients: When the CCR5 antagonist, the first reported gene associated with enhanced recovery was tested, and more significant recovery of cognitive function and neurological impairment was found. Apolipoprotein E epsilon four allele presence was studied in association with improved long-term outcomes in severe TBI patients. Nevertheless, no association was found [100].

An experiment that tested astaxanthin's role in reducing oxidative stress by targeting specific signal pathways found promising results in terms of neuroprotection and improving neurological function after TBI. The role of acetylcholinesterase inhibitors in pediatric and adult neuro recovery after TBI was evaluated with moderate effect sizes because of the small sample size [90,101].

Methylphenidate was also studied to test its safety and efficacy in improving complex attentional functions and processing speed, yet no benefit was concluded from this study. Another study of methylphenidate has found a modest improvement in attention, memory, and executive function after TBI, and additional research is needed.

Due to the role that oxidation and inflammation play in TBI and its link to poor outcomes, Enzogenol, a flavonoid-rich extract from Pinus radiata, was investigated and shown to improve cognitive functions in individuals with mild TBI [102].

Rivastigmine has been studied in veterans with a history of TBI, and neurotic deficit was hypothesized as a contributor to cognitive impairment; however, there were no significant differences compared to placebo [103].

The central neuropathic pain experienced after a traumatic injury to the spinal cord affects the quality of life of an individual drastically and hinders the rehabilitation process. Central neuropathic pain improved after a randomized clinical trial that utilized mirogabalin and demonstrated promising results with a decrease in neuropathic pain [104].

Furthermore, the use of dextroamphetamine, memantine, tranexamic acid, and citicoline was tested, but they failed to show any benefit and may require more studies to evaluate. Anti-inflammatory therapies, such as the recombinant interleukin one receptor antagonists hypothesis, were evaluated in terms of their potential newer rehabilitation benefits, but no controlled trials were conducted [105-107].

Neuromodulation

Noninvasive Brain Stimulation

Brain stimulation establishes neuromodulation, which leads to changes in function and structure. It creates either a magnetic field or electrical currents that stimulate targeted areas in the brain. For neuronal stimulation to be conducted effectively, an ion flow with a trans-membrane current must be directed outward. Changes in neurotransmitters, gene expression, and blood flow establish further neuroplasticity. Transcranial brain stimulation has been a very appealing non-pharmacological treatment due to its safety [108].

The role of noninvasive brain stimulation in TBI patients: It has been long believed that neuronal injuries represent a loss that can not be repaired. However, neuromodulation showcases the ability of neurons to adjust in terms of structure and function. It contributes to the possible role of its promising neuroplasticity results in neurorehabilitation. However, there has been controversy regarding TMS causing seizures in TBI patients. However, it was found that the link to seizure is related more to the severity of the brain injury rather than to the TMS intervention. Moreover, brain stimulation showed great potential in treating TBI-related motor injuries and post-TBI symptoms such as depression and neglect. A review conducted on the role of neuromodulation therapy in patients with a disorder of consciousness supports the ability of neuromodulation to improve DoC [109-112].

In both TDCS and TMS, as the neurons are stimulated, blood flow and metabolism changes occur. Such changes can be seen in multiple imaging modalities, and increased neuronal activity can be seen in electroencephalography. When experimented on rats, combined with rehabilitation training, electrical cortical stimulation ECS is more invasive compared to TDCS, as it includes placed electrodes directly inserted in the epidural space after craniotomy. However, ECS has shown superior results, faster recovery of motor function during the recovery period, and poor outcomes in the very early stage after a TBI [113].

NIBS can be utilized as a diagnostic approach that allows the chance to examine multiple parts of the nervous system and therapeutic approaches as a part of neurorehabilitation in TBI, spinal cord injury, stroke, and Multiple sclerosis.

Transcranial Magnetic Stimulation

Using this method, an electrical field is created by creating an electromagnetic field that targets specific cortical areas-avoiding the resistance that the scalp and skull can create. The stimulation is not carried out with the magnetic field on itself, as the name may imply, but rather the same as the traditional method of electrical stimulation. However, magnetic stimulation is more convenient as it is less painful than electrical stimulation. TMS with high frequency offers an excitatory effect to the neurons where excitation leads to long-term potentiation and low frequency with an inhibitory effect leads to long-term depression [108,114,115].

TDCS 

Anodes and cathode electrode pads apply an electrical current directly to the scalp. A current directed from the anode to the cathode is placed on the targeted cortex region with a contralateral placement. Compared with TMS, this current may not be as strong, creating an uncomfortable skin sensation. A systematic review has shown TDCS to be safe and effective in improving cognition and motor function in TBI patients, whether applied with other therapeutic agents or alone [116,117].

The Role of Invasive Brain Stimulation

Deeper structures and more invasive display of the brain structures, such as the epidural using a craniotomy, are required in invasive brain stimulation modalities such as deep brain stimulation (DBS) and epidural motor cortex stimulation (EMCS). With strategic planning and surgical site mapping, a DBS electrode is implanted into the target area. DBS can target deep structures such as the subthalamus, substantia nigra, internal and external palladium, and the striatum, which could greatly benefit patients with Parkinson's disease [118]. The thalamus and periventricular or periaqueductal gray matter are commonly studied in treating chronic refractory pain [119]. However, the availability of less invasive methods to control chronic pain has made invasive brain stimulation fall out of favor.

DBS

The role of DBS has long been studied and evaluated. The promising improvement and relief of symptoms in some neurological conditions, such as Parkinson's, made it appealing. Clinical trials of highly regular DBS stimulation subthalamic nucleus, pars interna of the globus pallidus, show improvement in motor function and postural instability in Parkinson's patients who did not respond to pharmacological treatment [120-122]. Nevertheless, DBS is ineffective in improving fine motor function needed in daily life activities when outcomes were measured with time and force characteristics used to perform a bimanual task that mimics the daily life activities performance [123].

In conclusion, advances in the role of technology, including artificial intelligence, VR, robotics, and computer interfaces, are all significantly impacting rehabilitation and improving the quality of life. Using VR for rehabilitation in TBI patients has improved gross motor skills and cognitive and executive functions, which have been reported with AI rehabilitation. Furthermore, it is expected that pharmacological rehabilitation will be beneficial. However, most pharmacological interventions did not result in any benefit when compared to the standard of care, and some needed further exploring and showed promising results. Moreover, DBS results are promising, especially in TDCS. However, convenience and safety are determinants when deciding on brain stimulation intervention.

Interdisciplinary approach and patient-centered care

One of the main reasons for the importance of a multidisciplinary team and interdisciplinary approach to TBI relies on the fact that post-concussion symptoms manifest differently in each patient, which creates the need for different evaluations in diagnosis and treatment. Nonetheless, when faced with different challenges in each of their primary fields of care, all healthcare professionals must be able to count on each other's expertise in order to overcome such obstacles and proceed with optimized patient care, as some cognitive tests cannot be performed with severe physical paralysis [124,125]. In contrast, therapy may be affected by speech impairment. The association of psychological, speech, physical, and occupational therapists is essential in overcoming specific obstacles in each therapeutic process [12]. According to a retrospective and prospective cohort of 172 American patients, occupational therapists (OT) were found to help identify and measure the impact of different neurological functions, accelerating and guiding the patient care process [126]. An interdisciplinary team can also include general, family, and sports physicians, neurologists, neuropsychologists, physiotherapists, speech therapists, nurses, surgeons, and others [127].

A systematic review conducted in Australia has found interdisciplinary intervention beneficial in all analyzed studies, emphasizing post-concussion symptoms such as mood, physical recovery, headaches, and cranial nerve function. Another case report study conducted in the USA has found similar results [128]. In this study, four patients with uncomplicated TBI were provided care by an interdisciplinary team consisting of a neuropsychologist, occupational, speech, and physical therapists, and social and vocational counselors. At the end of three months of weekly treatment, all four reported and exhibited decreased symptoms and increased self-sufficiency and life quality. Similarly, with more aggravated TBI also in the USA in a retrospective cohort of 60 patients, a care team, including physical, occupational, respiratory, and speech therapists, nurses, social counselors, and trauma surgeons, has helped decrease patient's hospitalization time (therefore leading to decreasing in cost), mortality and readmission rates. Even in adolescents, according to a randomized clinical trial conducted in Washington for six months, the addition of cognitive behavioral therapy to the usual care led to less neurological and psychological sequelae and an increase in well-being and quality of life in a one-year life span [129,130].

One of the most challenging parts of the TBI treatment sequence consists of post-hospitalization care and subsequent patient prognosis, including the part played by family and caregivers. TBI is frequently related to functional sequelae, which play an essential part in decreasing patients independence, well-being, and quality of life, as well as calls for a prolonged - and sometimes never-ending - recovery process. Communication between patients, caregivers, and medical staff is crucial for an adequate recovery process, especially regarding prospects for the future and recovery possibilities, where expectations must be aligned to provide the best possible care dynamic [131,132]. Additionally, properly instructing and teaching families and caregivers to provide adequate home care to TBI patients is a challenge that medical teams must face. Caregivers need to be educated about symptoms and their chronicity, implementation of new routine habits, factors that may aid or prejudice recovery, and proper ways to act accordingly [133]. Additionally, subsequent treatment plans, such as future appointments and consultations, eventual future needed surgeries, and physio or speech therapy sessions, must be planned carefully, and their importance should be adequately stressed to patients and caregivers, along with a progress plan and milestones to be reached. Other forms of support, such as social, emotional, and motivational, are also necessary and must be adapted to each patient and their caregiver's context, lifestyle, and possibilities. Seeing TBI patients as more than their brain injury aids in promoting patient-centered care and helps patients gain back a more individual and properly owned lifestyle [134]. It is important to remember that patients are not equal to their disease and once had a well-established and independent life before their TBI.

According to a study conducted by Rigney et al. [135], certain parental traits in various caregiver family profiles have been identified as significantly negative influences on children recovering from TBI [135]. They were found to be a family history of neuro or psychiatric injury, emotional issues such as concern, fearfulness, and tension, lower educational background, lower salary earnings, and already existing difficulties regarding the relationship between patients and caregivers before TBI. The authors recommend that staff consider these factors, as they play a direct part in the recovery process, and those in their possession might need extra assistance. Another systematic study has found similar traits related to prolonged care of TBI patients, such as caregivers' health conditions, abundance or lack of resources, a feeling of having a constant load to deal with, and prior quality of relationship with the patient. Stress in caregivers can include feelings of exhaustion, isolation, depression, overwhelmingness, and lack of control over their own lives [136,137]. Strengthening strategies for caregivers involved in TBI treatment can include teaching coping, management, and problem-solving skills regarding the actual care process and the issues directly related to the caregiver's emotional state. Such techniques could be addressed personally or online, assuring support even when the caregiver cannot always attend an in-person environment. These techniques improved treatment success and patient-family quality of life, especially at the beginning of home care.

Not only caregiver traits are analyzed when it comes to treatment success but also those of patients. Levels of depression, low self-esteem, and feelings of uselessness and of being a heavy load to their caregivers are often present in patients undergoing TBI recovery, especially when reaching the state of homecare, where most of the assistance is now transferred to the family [137]. It is, therefore, vital to proceed with a careful adaptation process of leaving the hospital and leading home, where both the patient's and caregivers' wants and needs must be considered [138].

Outcome measurement and evaluation in TBI 

Tools and Scales for Assessing Recovery and Functionality

In terms of assessing recovery after a TBI, multiple aspects could be affected, such as motor and cognitive function. Assessing recovery requires a multifaceted approach. Regarding assessing neuropsychiatric recovery, some commonly used tools are the general cognitive assessment tools such as mini-mental state examination. The Neuropsychological Assessment Battery (NAB) provides further aspects to be assessed: memory, attention, executive function, and assessment of language and speech.

Despite the high prevalence of impaired self-awareness in patients with moderate to severe brain injury, multiple validated tools are available to assess this condition [139]. No single golden standard measure has been identified to measure impaired self-awareness, with the most widely used tool being self-proxy discrepancy scores.

Challenges and future directions in TBI rehabilitation

Barriers to rehabilitation and recovery post-acute care are primarily associated with several factors, such as inadequate access to services, socioeconomic differences, and financial circumstances. A significant challenge continues to be the lack of availability and accessibility to rehabilitation services for TBI survivors. After acute treatment, most patients are discharged to nursing facilities that are not equipped to provide customized, comprehensive care and often even lack skilled specialists. Only about 13-25% of patients who survive moderate, severe, or penetrating TBI receive interdisciplinary inpatient rehabilitation, and even fewer receive TBI-specialized rehabilitation care [140]. Disparities in access to acute care and recovery services exist between rural and urban populations. The burden of TBI in terms of injury severity, outcomes, and survival is worse in rural areas than in urban areas [141]. Rural areas have fewer trauma care centers with insufficient resources, resulting in ineffective and substandard treatment. The severity of the injury, in combination with the lack of specialized care, doubles the impact of TBI in rural populations compared to urban groups.

Factors that influence recovery and outcomes after TBI are also underexplored. Sex-based differences in terms of injury severity, structural brain changes, and presentation of symptoms pose a knowledge gap in the facilitation of tailored treatment for both male and female populations [142]. Additionally, insurance coverage is a significant determinant that influences access to rehabilitation services. The individual's insurance status is strongly associated with recovery and outcome rates [143]. Due to rehabilitation's comprehensive and exhaustive nature, most inpatient and outpatient care centers require patients to be medically insured to avail of these services. The probability of receiving acute hospital care and post-acute rehabilitation is highly reduced due to the lack of insurance [143]. Moreover, patients and families have difficulty explaining their needs to insurance providers because of the imperceptible nature of cognitive communication difficulties, making it a more cumbersome process. Given the inequitable access to inpatient rehabilitation due to the lack of insurance, many patients are forced to seek services in community settings where navigating through the system is more challenging [144].

Although it is known that rehabilitation in the context of TBI is a demanding and extensive practice, with most patients requiring lifelong support through these interventions, follow-up from providers is often discontinuous. In addition, the current practices and systems for TBI care are fragmented, during which information can be lost [145]. The hierarchy of management for TBI is diverse, starting from acute care to rehabilitation interventions, which involves handing off the patient multiple times from one care setting to another, with the risk of disruption in care. This presents an opportunity for mistakes to be made.

Another crucial aspect of treatment initiation is an appropriate diagnosis of TBI, often challenged by the need for more sufficient criteria and terminology. The current classification of TBI into mild, moderate, and severe using the GCS can result in differing interpretations, which may introduce biases in care. The absence of universally accepted criteria to classify TBI is a significant roadblock in diagnosing and managing TBI [145].

Emerging trends and innovation

Research studies illustrate the need to identify the most effective interventions given the heterogeneity of TBI and the various circumstances under which it occurs in different population groups [145]. Moreover, future research must focus on developing guidelines for TBI management supported by evidence and should include the patient's perspectives on the current rehabilitation measures. The feedback and concerns of family members who are the patient's primary caregivers should be considered for building an effective treatment regime.

With advancements in neuroimaging techniques, future research can study or track neuroplastic changes in the brain to monitor cognitive improvements due to rehabilitation regimes [146]. Despite the potential of currently available blood-based biomarkers to enhance the diagnosis of TBI and provide a deeper insight into the underlying pathophysiology, the accessibility to these tools is limited [3]. Efforts must be made to expand the incorporation and utilization of biomarker tools for enhanced clinical decision-making regarding patient treatment. Given the high degree of variability in the causes, mechanisms, and injury severity of TBI across different population demographics and the involvement of multiple care providers, there is a need to integrate the current clinical data and build consensus-driven processes through systematic consolidation of information and coordination across hospital settings, acute care and rehabilitation centers, and community settings. Continued efforts to build strategies that enable brain-injured individuals to gain access to rehabilitation interventions are crucial to reducing the burden of disability caused by TBI [145].

## Conclusions

This narrative review has aimed to describe, outline, and explore strategies and outcomes for TBI recovery, especially regarding the role of technology, pharmacological treatments, and general interdisciplinary patient-centered care. Rehabilitation after TBI happens through neuroplasticity, the combination of neural regeneration - which involves growing new neurons and strengthening connections - and functional reorganization - in which the brain redistributes functions to compensate for damage. The role of technology, including artificial intelligence, virtual reality, robotics, and computer interface, are all making a significant impact in terms of rehabilitation and life quality improvement, as mainly virtual reality improved gross motor skills, and AI rehabilitation played a part in improving cognitive and executive functions. Pharmacological intervention, however, did not result in any benefit when compared to standard care and still needs further research. Lastly, the results obtained from DBS are promising, especially in TDCS. It is possible to conclude that, given the high and diverse degree of disability associated with TBI, it is essential to provide effective treatment and already initiate rehabilitation in the acute phase itself.
